# Review of Liquid Vitamin A and E Formulations in Veterinary and Livestock Production: Applications and Perspectives

**DOI:** 10.3390/vetsci11090421

**Published:** 2024-09-09

**Authors:** Yauheni Shastak, Wolf Pelletier

**Affiliations:** Nutrition & Health Division, BASF SE, 67063 Ludwigshafen am Rhein, Germany

**Keywords:** vitamin A, vitamin E, animal, requirement, veterinary, liquid, well-being

## Abstract

**Simple Summary:**

Vitamins A and E are crucial for animal health, contributing to key functions such as vision, immune response, growth, reproduction, and neurological development. A deficiency in these micronutrients can lead to serious health issues. Supplementation, especially with innovative liquid formulations, is important for addressing these deficiencies and enhancing animal health and productivity. Administering these vitamins through drinking water offers a convenient method, especially during times of stress or increased nutritional demands. Injectable forms can enhance reproductive health, growth, and immune function in livestock, while liquid drops provide a precise and easy way to dose companion animals. Future research may focus on optimizing these formulations, developing specialized treatments, and leveraging nutrigenomics to customize animal nutrition, leading to advancements in veterinary care and livestock management.

**Abstract:**

Vitamins A and E are vital fat-soluble micronutrients with distinct yet intertwined roles in various biological processes. This review delves into their functions, nutritional requirements across different animal species, the consequences of deficiencies, and the impact of liquid formulations on veterinary medicine and livestock production. Vitamin A exists in multiple forms, essential for vision, immunity, and growth, while vitamin E acts primarily as an antioxidant, safeguarding cell membranes from oxidative damage. Hypovitaminosis in these vitamins can lead to severe health consequences, affecting vision, immunity, growth, reproduction, and neurological functions. Hence, supplementation, particularly through innovative liquid formulations, becomes pivotal in addressing deficiencies and enhancing overall animal health and productivity. Injectable forms of vitamins A and E show promise in enhancing reproductive performance, growth, and immune function in livestock. Administering these vitamins through drinking water offers a convenient way to enhance livestock health and productivity, particularly during times of stress or increased nutritional needs. Liquid vitamin A and E drops offer a flexible and effective solution in veterinary practice, allowing precise dosing and easy administration, particularly for companion animals. Future research may aim to optimize formulations and explore targeted therapies and precision feeding via nutrigenomics, promising advancements in veterinary medicine and livestock production.

## 1. Introduction

There are thirteen vitamins (excluding choline) that are used in animal nutrition. From a commercial perspective, vitamin E was the most consumed feed-grade vitamin in 2022, with an estimated market value of around half a billion USD. This was followed by B-group vitamins and vitamin A [1]. Thus, vitamins E and A can be regarded as the most commercially significant fat-soluble vitamins and among the most commercially relevant of all vitamins.

Vitamins A and E are essential micronutrients crucial for the health and performance of animals [2,3]. They are typically included in the diets of both ruminant and monogastric animals through premixes. Though they have distinct biochemical properties—retinoic acid signaling and the visual cycle for vitamin A, and antioxidant defense and membrane stabilization for vitamin E—their functions are interconnected, collectively supporting various bodily systems. For instance, vitamin E regulates redox balance by modulating the NFκB/AKT/mTOR/KEAP1 signaling pathway and NRF2 activation, supports liver histoarchitecture restoration by enhancing oxygen uptake under various stress conditions, and is also suggested to influence the promoter-specific activity of redox-responsive genes [4,5]. As fat-soluble vitamins, they are crucial for modulating immune responses, supporting reproductive functions, and maintaining cellular integrity [6,7,8,9,10]. Deficiencies in these vitamins can lead to numerous health problems, affecting animal welfare and productivity. Liquid formulations of vitamins A and E have been effective alternatives to dry forms in veterinary medicine and livestock production for decades, offering benefits such as precise dosing, ease of administration, and improved bioavailability [11].

Ensuring the well-being and productivity of domesticated animals requires meeting their vitamin A and E requirements, as they cannot synthesize these micronutrients metabolically de novo. The inability to synthesize a vitamin typically arises from the absence of genes responsible for its synthesis, a trait that emerged during the course of evolution [12]. Therefore, their dietary intake or other supplementation routes are crucial. The required level of vitamins A and E in animals depends on several factors, including physiological state, age, health, nutritional status, and function [10]. Understanding the nutritional requirements and factors affecting the intake of vitamins A and E across different animal species is crucial for optimizing health and productivity [3,10,13,14,15,16]. For instance, ruminants, with their complex digestive systems, require careful management of supplemented vitamin levels due to potential losses or degradation in the rumen, particularly for vitamin A [17]. Similarly, the diets of growing monogastric animals, such as poultry and swine, are often pelleted, which can lead to additional vitamin loss [15,18]. Ultimately, the key factor is not the initial amount of vitamin A intended to be supplied to the animal, but how much of this vitamin is actually reaching the animal and how bioavailable the vitamin form is.

Liquid formulations of vitamins A and E, as well as their combinations with other vitamins and active substances, have garnered attention for their enhanced bioavailability and ease of administration, offering some advantages over traditional dry forms. These liquid supplements, commonly utilized in veterinary medicine and livestock production, provide the quick and efficient delivery of essential micronutrients, especially during periods of stress or increased demand [19]. Injectable forms have shown promising results in enhancing reproductive performance and animal growth, with studies demonstrating positive effects on litter size in swine, muscle development, and marbling in beef cattle [20,21,22,23,24,25].

Administering liquid forms of fat-soluble vitamins through water has emerged as a practical strategy for enhancing livestock health and productivity, particularly during periods of illness or stress [11]. Liquid vitamin A and E drops are widely used in veterinary practice, offering a convenient means of addressing deficiencies and promoting overall well-being in companion animals [11,26].

This review aims to explore the role of liquid vitamin A and E in veterinary and livestock production. Despite extensive research on vitamins A and E, there is a notable gap in understanding the comparative effectiveness and specific applications of liquid formulations across different animal species. We hypothesize that liquid vitamin A and E formulations may offer distinct advantages over traditional supplementation methods, including enhanced bioavailability and practical benefits.

Ultimately, this review seeks to enhance our understanding of the role of liquid vitamin A and E in veterinary and livestock practices. Such knowledge can guide the development of more effective management strategies to address deficiencies, improve stress resilience, and enhance overall animal well-being and productivity.

## 2. Roles and Nutritional Needs across Animal Species

Vitamins A and E are vital fat-soluble micronutrients. They play key roles in various biological processes crucial for cellular health and overall physiological well-being.

### 2.1. Vitamin A

**Figure 1:**
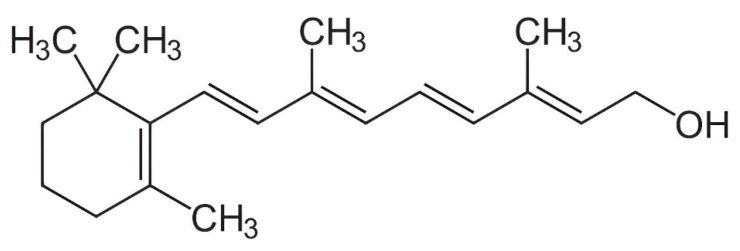
Structural formula for all-trans-retinol.

Vitamin A includes compounds like all-trans-retinol, retinal (retinaldehyde), and all-trans-retinoic acid (ATRA), each essential for different bodily functions [27]. Retinol, primarily stored in the liver, can be converted into retinal and ATRA as needed [10] (Figure 1). Retinal is vital for vision, especially in low-light conditions, as it combines with opsin proteins in the retina to form rhodopsin, a pigment critical for phototransduction [28].

**Figure 2:**
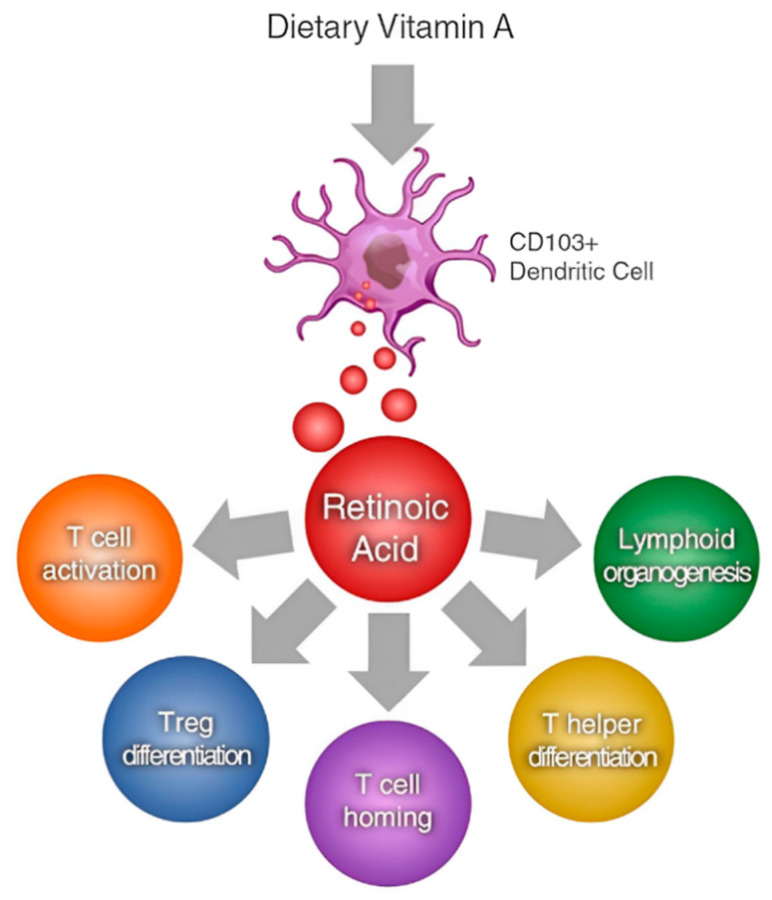
ATRA (all-trans-retinoic acid) as a modulator of T cell immunity [36].

Beyond vision, vitamin A is crucial for cellular differentiation and proliferation. ATRA, the active metabolite of retinol, regulates gene expression by binding to nuclear receptors, controlling the transcription of genes involved in cell growth, differentiation, and apoptosis [29,30,31,32]. This regulation is vital for maintaining epithelial tissues such as the skin, respiratory tract, and gastrointestinal lining [33,34]. Additionally, vitamin A enhances immune function, supporting T cells, B cells, and natural killer cells, while maintaining mucosal barriers [35] (Figure 2).

Vitamin A absorption begins in the intestine, where dietary retinyl esters are hydrolyzed to retinol [10]. Retinol is absorbed by enterocytes, re-esterified, and transported to the liver [37]. In the liver, retinol is stored as retinyl esters and mobilized when needed [38].

The accurate measurement and utilization of different forms of vitamin A are crucial for proper dosages in commercial products. The units of measurement for vitamin A are as follows:

- 1 IU of vitamin A is equivalent to
  ○ 0.3 μg of retinol (vitamin A alcohol);
  ○ 0.344 μg of retinyl acetate (vitamin A acetate);
  ○ 0.359 μg of retinyl propionate (vitamin A propionate);
  ○ 0.55 μg of retinyl palmitate (vitamin A palmitate).

### 2.2. Vitamin E

**Figure 3:**
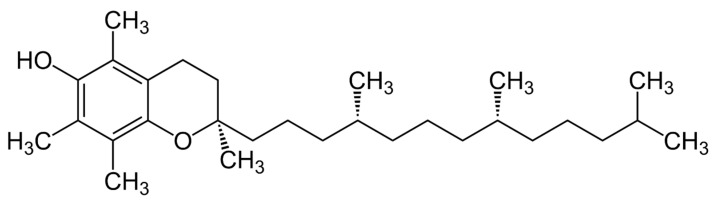
Structural formula for α-tocopherol.

Vitamin E encompasses eight related compounds, including four tocopherols and four tocotrienols, with α-tocopherol being the most biologically active and abundant in animal tissues (Figure 3). Vitamin E is renowned for its antioxidant properties, protecting cell membranes from oxidative damage [39]. It donates a hydrogen atom to free radicals, neutralizing them and preventing lipid peroxidation within cellular membranes [40]. This antioxidant capability is crucial for maintaining cell membrane integrity, especially in oxygen-exposed tissues like the lungs, liver, muscle, and red blood cells [41].

Vitamin E also influences gene expression and immune function by modulating enzymes involved in cell signaling and regulating genes related to antioxidant defense, inflammation, and cell cycle regulation [42,43,44,45]. For example, vitamin E can inhibit protein kinase C, a kinase involved in cell proliferation and differentiation, thereby exerting anti-inflammatory effects [46].

The absorption of vitamin E occurs in the small intestine, where it is incorporated into chylomicrons and transported via the lymphatic system to the bloodstream [47]. Like vitamin A, vitamin E is stored in adipose tissue and the liver.

The standardized units for measuring vitamin E are as follows:

- 1 mg of DL-alpha-tocopheryl acetate is equivalent to 1.0 IU of vitamin E;
- 1 mg of D-alpha-tocopheryl acetate is equivalent to 1.36 IU of vitamin E.

### 2.3. Interplay between Vitamins A and E

Vitamins A and E interact synergistically to maintain cellular health. For instance, α-tocopherol enhances the antioxidant efficacy of all-trans-retinol by reducing its autooxidation, providing synergistic protection against peroxidative stress [48,49]. Additionally, vitamin E spares vitamin A by protecting it from oxidation, increasing its intestinal absorption, and enhancing its storage [50]. Studies have shown that animals fed vitamin E had a tenfold higher liver vitamin A content than those without supplementation [51]. However, excessive intake of either vitamin can negatively influence the storage levels of the other [26].

### 2.4. Nutritional Requirements in Different Animal Species

The nutritional needs of vitamins A and E vary among species due to differences in digestive systems, gut microbiota, dietary composition, and physiological needs [10,13,14,52]. Factors such as age, health status, and overall nutritional status also affect these requirements.

Ruminants: Ruminants, like cattle, have complex digestive systems that may lead to potential vitamin A and E loss or degradation [53,54]. While ruminants can convert β-carotene from forage into vitamin A, supplementation with retinyl acetate and DL-α-tocopheryl acetate is often necessary to meet their requirements [14,55,56]. The National Academies of Sciences, Engineering, and Medicine (NASEM) provide “Adequate Intake” levels for these vitamins based on the available research (Table 1 and Table 2) [17,57,58,59]. Furthermore, other scientific organizations, including the German Society of Nutrition Physiology (GfE), the National Institute of Agricultural Research (INRA), the Ministry of Agriculture of the People’s Republic of China (MOA), and the Centraal Veevoederbureau (CVB), also offer foundational guidelines for formulating ruminant feed [60,61,62,63].

Monogastrics: In monogastric animals like poultry, swine, and companion animals, vitamins A and E are standard dietary supplements. Carnivores like dogs and cats require animal-derived retinoids due to their limited ability to convert β-carotene to retinal [64,65,66,67,68]. Poultry and swine also require supplementation due to their inefficient conversion rates of ß-carotene and the variability of ß-carotene levels in their diet [69,70]. Optimal vitamin A and E supply recommendations are presented in Table 3.

## 3. Consequences of Deficiencies in Vitamins A and E

Deficiencies in vitamins A and E have profound and overlapping impacts on health in animals, as these vitamins are crucial for various physiological functions, including vision, immune function, growth, reproduction, and protection against oxidative damage.

Ocular Health: Vitamin A is essential for vision, primarily due to its role in rhodopsin, a pigment necessary for low-light vision. In cattle, a deficiency of vitamin A commonly results in night blindness and xerophthalmia [15]. Similarly, in poultry, vitamin A deficiency can cause conjunctivitis, corneal ulcers, and excessive tearing, potentially progressing in severe cases to blindness [14,84,85,86]. In pigs, a lack of vitamin A may lead to visual impairment and, in extreme cases, complete blindness [10]. In felines and canines, deficiency symptoms include night blindness, reduced vision in dim light, conjunctivitis, dry eye with inflammation, corneal neovascularization, light sensitivity, dilated pupils in normal lighting, delayed pupillary light reflex, progressive retinal degeneration, cataract formation, and, potentially in severe cases, blindness [67,87,88]. Vitamin E, with its antioxidant properties, helps protect ocular tissues from oxidative damage.

Immune System: Vitamin A plays a crucial role in maintaining mucosal barriers and supporting the differentiation of immune cells [2]. In cattle, vitamin A deficiency is associated with increased susceptibility to infections, particularly respiratory and gastrointestinal diseases, due to compromised immune function [15]. In swine, deficiencies in both vitamins A and E weaken immune responses, leading to increased vulnerability to infections [3,10]. Similarly, in poultry, deficiencies in these vitamins compromise the integrity and function of various immune cells, reducing their ability to combat infections [3,9,14]. For carnivorous companion animals such as dogs and cats, deficiencies in vitamins A and E lead to impaired immune function and a higher predisposition to infections [67].

Growth and Development: Sufficient levels of vitamins A and E are crucial for proper growth and development. In growing cattle, vitamin A deficiency is linked to stunted growth and skeletal abnormalities [15]. In poultry, deficiencies in both vitamins impair neuromuscular development, leading to conditions such as ataxia, peripheral neuropathy, and white muscle disease [3,14]. In swine, a lack of vitamin A negatively impacts growth and development, while insufficient vitamin E reduces growth performance [10,89,90,91].

Reproductive Health: Both vitamins are essential for reproductive health. In dairy cattle, vitamin A deficiency can result in a reduced sperm count and motility in males, as well as impaired ovarian function and embryonic development in females, potentially leading to infertility and complications during pregnancy [15,92,93]. Vitamin E supports reproductive health in sows, where deficiencies can cause reproductive failure and increased neonatal mortality [3,94]. Historically, vitamin E has been referred to as the “anti-sterility vitamin” due to its significant role in reproduction [95] (Table 4).

In domestic fowl, inadequate vitamin A levels can decrease egg production, lower hatchability rates, and increase embryonic mortality [14,96]. Reproductive complications, such as infertility or dystocia, can also occur due to deficiencies in these vitamins in dogs and cats [67,97].

Combined Impact: Deficiencies in both vitamins, A and E can have a synergistic detrimental effect. Together, they significantly impair immune function, growth, development, and reproductive health. Both vitamins, or their derivatives, either act as antioxidants or stimulate the production of antioxidative enzymes; thus, their combined deficiency increases oxidative stress, leading to cellular damage and a heightened risk of chronic diseases [98,99,100].

## 4. Liquid Vitamin A and E Supplements

Liquid vitamin supplements have gained considerable attention due to their enhanced bioavailability and ease of administration compared to traditional dry forms [101,102]. Vitamins A and E, essential for numerous physiological functions, are commonly formulated into liquid supplements to address or treat specific dietary deficiencies and promote overall health during stress periods in animals [103].

According to the EFSA [104], in the preparation of liquid formulations, retinyl esters that already include antioxidants/stabilizers are uniformly combined with a stabilizer and, optionally, with a liquid feed grade carrier to regulate the concentration of the active substance. For instance, liquid (oily) formulations of vitamin A such as acetate, palmitate, and propionate may have a concentration of 0.25 to 2.5 million IU per gram, and may contain carriers (e.g., plant oil 3–60%) and antioxidants (e.g., butylhydroxytoluene (BHT), butylhydroxyanisole (BHA), DL-alpha-tocopherol, and ascorbyl palmitate, 1–25%).

### 4.1. Stability and Formulation Strategies

A significant challenge associated with vitamin A is its susceptibility to degradation. Factors such as humidity, oxygen, heat, light, and heavy metals can compromise its stability [105]. To mitigate this issue, many vitamin A formulations incorporate antioxidants [104]. However, research has shown that employing a dry inert gas to prevent exposure to oxygen and moisture can maintain the stability of pure retinyl palmitate and retinyl propionate, regardless of the presence of an antioxidant [11]. In contrast, DL-α-tocopheryl acetate, commonly used in liquid vitamin preparations, stands out due to its resistance to oxidation, unlike α-tocopherol and vitamin A esters, making it one of the stable vitamins [106].

Liquid vitamin A supplements for veterinary use are commonly formulated as retinyl palmitate or retinyl propionate, both of which are esterified forms of retinol. These formulations are preferred for their stability and bioavailability [107]. Retinyl palmitate and retinyl propionate are often favored over retinyl acetate due to their greater stability in solution, especially at higher temperatures [108]. In fact, retinyl acetate is rarely used in liquid formulations because it tends to crystallize out of oily solutions and is more difficult to solubilize compared to retinyl palmitate and retinyl propionate [11].

The solubilization of fat-soluble vitamins often involves creating micelles using non-ionic surfactants, known as solubilizers, composed of lipophilic and hydrophilic components [109]. These micelles trap molecules like vitamin A and E, making them imperceptible if not overloaded. However, as they grow larger, the solution may appear opalescent [11].

In practice, solubilization requires heating the mixture of vitamin and solubilizer with an antioxidant in the absence of water [110]. Water should only be added once the mixture reaches 60–70 °C, or vice versa, while stirring vigorously. Deviating from this process often results in turbid or opalescent solutions [11].

### 4.2. Liquid Formulations of Vitamins A and E

Various liquid forms of vitamins A and E or their combination with other vitamins are currently available:

- Oil-based solutions: Oil-based formulations often use vegetable oils, such as sunflower oil, as carriers for vitamins [111,112]. These formulations may also incorporate hydrophobic solvents like short-chain saturated triglycerides, ethyl stearate, or liquid paraffin [11]. A high-quality oil, especially one rich in α-tocopherol, can enhance the stability of vitamin A by protecting it from oxidation and improving its absorption in the gastrointestinal tract [113]. To further stabilize these formulations, compounds like BHT are frequently added, particularly in products containing retinyl propionate or retinyl palmitate [114]. It is important to note that vitamin A tends to be more stable in oily solutions compared to aqueous ones [11]. In these oil-based products, vitamin E acetate can be included either in its undiluted form or mixed with oils.
- Emulsions: Emulsions are a versatile and widely used drug formulation designed to enhance the solubility and bioavailability of vitamins, particularly for lipophilic vitamins such as A and E. These formulations are especially beneficial in aqueous environments where solubility can be challenging. The effectiveness of emulsions is often due to the creation of transparent microemulsions, which significantly improve the solubility and stability of these vitamins [110,115].
  These emulsions are particularly advantageous for species with less efficient fat digestion or in scenarios where rapid vitamin absorption is required. For instance, a standard emulsion product may contain 50,000 IU/mL of vitamin A, emulsified with lecithin and polysorbate 80 [11]. This combination creates a stable and homogeneous mixture, which ensures effective delivery and absorption of the vitamin. Polysorbate 80, a non-ionic solubilizer commonly used in both oral and topical pharmaceuticals, has hydrophobic and hydrophilic components. The primary hydrophobic part in polysorbate 80 is polyethylene glycol-20 sorbitan oleate, which plays a crucial role in the solubilization process [116].
  Another example of an emulsion is a vitamin A/D/E preparation for injection, which includes 23.0 g of retinyl propionate, 0.2 g of cholecalciferol, 5.5 g of DL-α-tocopheryl acetate, 15.0 g of PEG-15 hydroxystearate (as a solubilizer), 0.5 g of butylated hydroxytoluene, 1.0 g of benzyl alcohol, and water to make up 100 mL [11].
  A specialized type of emulsion is the microemulsion, where retinyl esters, for example, are emulsified in an aqueous solution containing substances such as gelatin and sugar [104]. Microemulsions consist of droplets of the oily phase, approximately 1 μm in diameter, and remain stable within a temperature range of 50–60 °C [115]. Additionally, various types of fat-soluble vitamin emulsions can be formulated, including oil-in-water, water-in-oil, and oil-in-water-in-oil emulsions, offering a range of options depending on the desired application [117].
- Aqueous Solutions: To effectively produce aqueous solutions of lipophilic vitamins such as A, D, E, and K, the use of solubilizers is essential. Key solubilizers include Polysorbate 80, PEG glyceryl trihydroxystearate, PEG glyceryl triricinoleate, and PEG hydroxystearate, all of which are known to form micelles that encapsulate these lipophilic vitamins, thereby facilitating their dissolution in water [11]. The quantity of solubilizer required can vary considerably depending on the specific vitamin. For instance, the solubilizer needed for vitamin E acetate differs from that required for vitamin A.
  Specific formulations can be used to prepare clear aqueous solutions, as detailed by Buehler [11]. For example, to produce unstabilized vitamin A drops (50,000 IU/mL), the formulation may include 3.0 g of retinyl palmitate, 10.0 g of PEG 40 glyceryl trihydroxystearate, 5.0 g of polyethylene glycol 400, and 100 mL of water. A vitamin E acetate solution (20 mg/mL) might consist of 2.0 g of DL-α-tocopheryl acetate, 8.0 g of polysorbate 80, and 100 mL of water. Additionally, a formulation for combined vitamin A and E drops (825,000 IU + 50 mg per ml, respectively) could use 1.50 g of retinyl palmitate, 5.0 g of DL-α-tocopheryl acetate, and 20.0 g of PEG 40 glyceryl trihydroxystearate, along with an appropriate amount of antioxidant, preservative, and flavoring, all dissolved in 100 mL of water. It is important to note that when using solubilizers, the effectiveness of preservatives must be carefully evaluated, as solubilizers can potentially diminish their efficacy.

## 5. Comparative Bioavailability

The method of administering vitamin A significantly influences its biological availability in animals. For example, injecting retinyl propionate directly into the bloodstream achieves a bioavailability rate of 100%. In contrast, other methods such as intramuscular (i.m.) or subcutaneous (s.c.) injections and oral supplementation via feed or water exhibit lower bioavailability [118]. The direct bloodstream injection of retinyl esters avoids the barriers and tissues encountered with other administration routes, resulting in quicker and more complete utilization [10]. In s.c. or i.m. injections, retinyl esters must diffuse through cellular barriers and blood vessels to enter systemic circulation. When administered orally, vitamin A must pass through the digestive system, where retinyl esters are broken down by digestive enzymes, absorbed across the intestinal wall, and metabolized in the liver before reaching systemic circulation [37]. This multistep process can lead to a lower biological value for orally supplied retinyl esters compared to injection methods [10]. The same rules apply for DL-α-tocopheryl acetate. Generally, i.m. injections have a quicker onset of action than s.c. injections due to the higher vascularity and blood flow in muscle tissue [119]. However, s.c. injections may provide a more prolonged duration of action because the active substance is absorbed and released more gradually from subcutaneous tissue [120]. It is also important to consider that different retinyl esters may have unique absorption characteristics depending on their specific properties, such as molecular weight and stability, as well as the injection site [10].

Brief and Chew [121] carried out an experiment with gilts that received either vitamin A supplementation through their diet or vitamin A propionate injections for four weeks prior to farrowing. The results showed that the injected gilts experienced reduced embryonic mortality and yielded a higher number of heavier piglets per litter compared to those that received dietary supplementation or were vitamin A deficient. This indicates that injectable vitamin A supplementation shows a higher biological value than dietary supplementation for enhancing reproductive outcomes in gilts.

Evaluating the bioavailability, or the degree to which a substance can be absorbed and utilized by the body, is crucial for lipophilic vitamins [122]. The presence of various additives, such as solubilizers, solvents, and other additives, can significantly impact the bioavailability of these vitamins [11].

**Figure 4:**
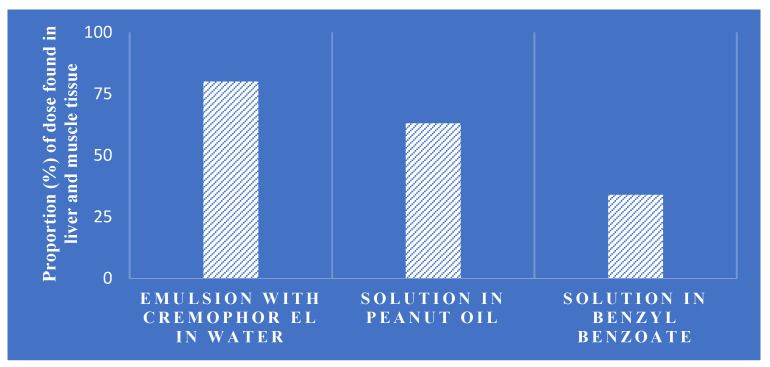
Vitamin A bioavailability in chickens 14 days post-parenteral administration [11].

A study conducted on chickens demonstrated the effect of these auxiliaries on the bioavailability of vitamin A solutions administered through injection. Figure 4 illustrates the variation in vitamin A retention observed when different additives were used in the solutions.

Thus, the choice of administration method and formulation significantly impacts the bioavailability and efficacy of lipophilic vitamins like vitamins A and E. Careful consideration of factors such as the injection site, the molecular properties of the vitamin esters, and the presence of auxiliaries is crucial to optimize the delivery and utilization of these essential micronutrients.

## 6. Applications in Veterinary Medicine and Livestock Production

When rapid supplementation of vitamin A and E is needed, liquid forms are preferred because they can be quickly supplied via water or injection. In contrast, using dry forms through feed supplementation requires adjusting the premix formulation and involves a lengthy feed production and delivery process. Unlike dry forms, liquid forms stored on the farm can be applied immediately. This chapter explores the roles and benefits of liquid vitamin A and E in enhancing animal health and reproduction, improving production parameters, and preventing diseases in various livestock species.

### 6.1. Use of Injectable Forms for Enhancing Reproductive Performance

One major challenge to profitability in the pork industry is the reproductive efficiency of breeding sows [10]. Vitamin A supports the reproductive system and fetal development [92,93,94,95,96,97].

Coffey and Britt [123] showed that injecting retinyl palmitate into sows already on a diet with adequate vitamin A (11,000 IU/kg of feed) increased litter sizes. Their treatment involved intramuscular administration of 50,000 IU of vitamin A at weaning, mating, and 7 days after mating, resulting in more piglets being born alive and a greater litter weight, highlighting retinol’s role in embryonic development.

Silveira et al. [124] conducted a field trial with 1030 sows, injecting 450,000 IU of retinyl palmitate at weaning, mating, and 7 days after mating. This treatment improved reproductive performance, increasing the number of live-born piglets and litter weight, leading to a recommendation of 500,000 IU of retinyl palmitate at weaning or 5 days before gilts’ expected heat.

Lindemann et al. [20] confirmed these findings, showing that injections of 250,000 IU or 500,000 IU of vitamin A at weaning and breeding in sows already on adequate diets consistently increased the litter size and weight. They concluded that vitamin A positively affects oocyte maturation, ovulation, fertilization, and early embryonic survival and development.

Whaley et al. [125] administered 1,000,000 IU of retinyl palmitate on day 15 after the second estrus to gilts on high-energy diets. This altered oocyte and embryo development, suggesting vitamin A influences embryonic development by advancing meiosis resumption. While many studies indicate vitamin A enhances sow reproductive performance, some report no effect, underlining the need for further research [126,127].

Similarly to vitamin A, vitamin E, also known as the anti-sterility vitamin, can effectively enhance reproductive performance. Pontes et al. [22] investigated the impact of injectable vitamin E administered during the last three weeks prepartum on retained fetal membranes (RFMs) and overall reproductive performance in dairy cows. In this study, 890 cows were randomly assigned to either a control group or a treatment group. The treatment group received three injections of 1000 IU of vitamin E before calving. The findings indicated that cows receiving vitamin E experienced a significant reduction in the incidence of RFMs, stillbirths, and levels of cortisol and non-esterified fatty acids around calving. Additionally, these cows had improved pregnancy rates compared to the control group. Notably, milk production was similar between the groups, but the treated cows demonstrated enhanced reproductive outcomes.

In another study, Mahmoud et al. [21] explored the effects of vitamin E and selenium injections on semen quality, testes measurements, and blood parameters in Ossimi rams. Fourteen rams were divided into control and treatment groups, with the treatment group receiving injections of 5 mg sodium selenite and 450 mg vitamin E twice weekly for a month. The treated rams exhibited significant improvements in semen quality and quantity, including increased ejaculate volume, mass motility, and sperm concentration, along with a reduction in dead and abnormal spermatozoa. Furthermore, the treated rams showed elevated serum testosterone levels and enhanced reproductive performance, underscoring the beneficial effects of vitamin E and selenium injections. Studies on female sheep have also demonstrated the positive impact of vitamin E injections on reproductive performance [25].

### 6.2. Use of Injectable Forms in Enhancing Animal Growth and Performance

**Figure 5:**
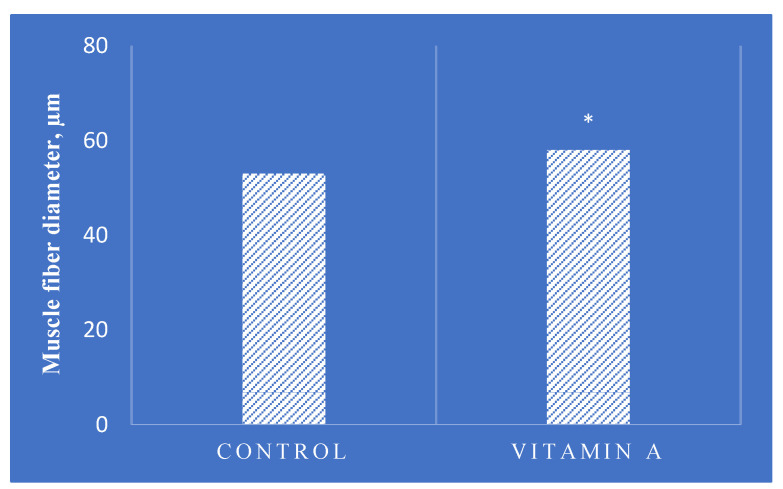
Average latissimus dorsi muscle fiber diameter at harvest (adapted from Wang et al., [23]). Angus steer calves were administered with 0 (control) or 150,000 IU vitamin A (retinyl palmitate) per calf at birth and 1 month of age. The resulting steers were harvested at 14 months of age. * *p* < 0.05; Mean, *n* = 9.

Vitamin A is essential for the growth and development of animals. Retinoic acid signaling significantly influences myogenic differentiation in mammals [128]. According to Wang et al. [23], injecting vitamin A into cattle enhances their growth (Figure 5) by boosting the number of PAX7-positive satellite cells and increasing the expression of myogenic marker genes, such as PAX7, MYF5, MYOD, and MYOG. Consequently, early-stage treatment with retinyl palmitate results in larger muscle fiber sizes in vitamin A-treated cattle at harvest. The researchers emphasized the importance of carefully managing vitamin A levels in beef cattle to optimize growth performance.

Harris et al. [24] discovered that providing vitamin A through injection to newborn beef cattle, at doses of either 150,000 IU or 300,000 IU (as retinyl palmitate), both at birth and one month of age, led to increased weaning weights. This enhancement was attributed to improved muscle growth and augmented marbling fat production, as detailed in Table 5. Consequently, incorporating vitamin A supplementation into the early developmental stages could serve as a feasible strategy for beef cattle producers seeking to enhance marbling and optimize beef production efficiency.

Similar outcomes were reported in lambs, where 16 newborn lambs received weekly intramuscular injections of either corn oil (control group) or 7500 IU vitamin A palmitate (vitamin A group) from birth to 3 weeks of age [129]. At 3 weeks of age, muscle samples were taken to analyze the effects of vitamin A. All lambs were slaughtered at 8 months of age. The results suggest that vitamin A treatment accelerated the growth rate, increased the loin eye area (*p* < 0.05), and increased the diameter of myofibers in the longissimus thoracis muscle (*p* < 0.01). Additionally, vitamin A increased the final body weight of lambs (*p* < 0.05). A recent trial showed that retinoic acid promotes lamb muscle growth by inhibiting myoblast proliferation and promoting myogenic differentiation through class E basic helix-loop-helix protein (40BHLHE40), a transcriptional repressor, which inhibits the expression of DNA-binding inhibitor 3 (ID3) [130].

In contrast to the research on the effects of vitamin A injection, there is a paucity of studies investigating the administration of vitamin E in growing animals and its consequent impact on performance. Thus, there is a compelling need for further experimental exploration in this area to elucidate its potential benefits or limitations.

### 6.3. Incorporating Liquid Vitamin A and E into Veterinary Practices via Water Administration

Administering liquid forms of fat-soluble vitamins through water has been an effective strategy for enhancing livestock health and productivity for decades. Supplementing these vitamins via water, particularly during periods of stress or increased demand, helps livestock achieve optimal physiological functions, potentially improving growth rates, reproductive performance, and disease resistance [8,131]. During illness or stress, animals typically reduce their feed intake but continue to consume water, providing an opportunity to supply essential micronutrients [132]. This method ensures the uniform distribution and efficient uptake of vitamins.

Vitamin A, D_3_, and E supplementation via drinking water is commonly used to support animals during periods of high demand. Schmidt [19] conducted a trial on 41 sows around weaning, dividing them into two groups. The trial group received 10 mL of a vitamin A, D_3_, and E combination in their drinking water from one day before until two days after weaning. The results showed that vitamin supplementation significantly shortened the weaning-to-estrus interval, with almost 50% of the sows returning to estrus within four days post-weaning. This indicates the positive impact of vitamin supplementation on reproductive function.

Amazan et al. [133] investigated the impact of vitamin E (α-tocopherol) supplementation in drinking water on tocopherol transfer, antioxidant capacity, and immune response in sows and piglets. Piglets receiving vitamin E in their water, whose mothers were also supplemented, exhibited the highest serum α-tocopherol levels five days post-weaning. The Ferric Reducing Ability of Plasma (FRAP) significantly improved with vitamin E supplementation (*p* = 0.037). However, there were no significant changes in immunoglobulin levels in piglet serum. These findings suggest that vitamin E supplementation through water enhances plasma antioxidant capacity, as demonstrated by the FRAP assay.

### 6.4. Usage of Liquid Vitamin A and E Drops in Veterinary Practice

Liquid drops containing vitamins A and E are widely utilized in veterinary medicine for a range of purposes, such as addressing deficiencies, enhancing overall well-being, and bolstering specific bodily functions, especially in companion animals [26]. The dosage and administration of these drops in veterinary practice typically hinge on factors like the animal’s age, weight, and particular health condition, with the veterinarian overseeing the process. It is crucial to adhere closely to the veterinarian’s guidelines and avoid surpassing the recommended dosage. These liquid drops can be seamlessly incorporated into the animal’s feed or administered directly into their mouth, offering added convenience for both pet owners and veterinarians, especially when dealing with animals that struggle with swallowing pills or capsules [134,135].

Liquid vitamin A drops can be utilized to treat various ailments. Silver [26] identifies several diseases in dogs and cats commonly associated with vitamin A deficiency, which can be alleviated or managed with retinoid supplements, including retinoid-responsive dermatoses, solar dermatosis of dogs and cats, xerophthalmia, squamous cell carcinoma, intestinal inflammation, keratoacanthoma, alopecia, delayed wound healing, sebaceous gland disorder, canine icthyosis, feline muzzle folliculitis, nyctalopia, keratoconjunctivitis sicca, squamos metaplasia, epitheliotrophic T cell lymphoma, respiratory infections, hyperkeratinization of the epithelial surfaces, seborrhea, haircoat problems, Schnauzer comedo syndrome, increased susceptibility to infection, exfoliation, sebaceous adenitis, and follicular dysplasia.

A recent study by Zhang et al. [136] demonstrated that supplementation with an oil-based oral vitamin A solution, at a calculated dosage of 8000 IU per kg of diet, enhanced the immune response in White Leghorn chickens infected with infectious bronchitis virus (IBV). The chickens received daily vitamin A supplementation until they were 21 days old, after which they were infected with a pathogenic IBV strain. The results showed that vitamin A supplementation reduced viral replication and increased serum IgG levels, while also mitigating the inflammatory response. Although the clinical course of the disease and the growth performance of the chickens were not significantly affected, these findings underscore the essential role of vitamin A in modulating chicken–IBV interactions and supporting innate immunity.

The evidence suggests that vitamin E possesses anti-inflammatory and analgesic properties [137,138]. In a study using a canine model of osteoarthritis, two groups of dogs were evaluated: one group received a placebo (n = 8), while the other group received 400 IU of liquid DL-α-tocopheryl acetate daily (n = 7) [139]. After 56 days, synovial fluid analysis revealed significantly lower concentrations of prostaglandin E2 and nitrogen oxides in the vitamin E-supplemented group. Assessments of pain and inflammation demonstrated lower scores in the vitamin E group, with significant reductions in visual analog scale scores by day 55 and in electronic von Frey scores by day 28. This study is the first to show that high-dose vitamin E supplementation can reduce inflammation and pain markers in dogs with osteoarthritis.

### 6.5. Regulatory Guidance

When using commercial forms of vitamins A and E in animal production, it is essential to comply with local regulatory requirements and usage conditions. The safe and appropriate application of vitamin A in particular necessitates adherence to specific guidelines. These guidelines limit vitamin A supplementation to the recommended intake levels set by authoritative bodies, such as the European Commission in the European Union, based on scientific opinions from the European Food Safety Authority (EFSA). Therefore, any use of vitamin A in animal nutrition must conform strictly to the regulatory framework to avoid excessive supplementation and potential adverse effects on animal health and welfare.

For instance, in the European Union, vitamin A used in water must follow specific guidelines. The combined concentration of vitamin A in water and feed must not exceed the recommended intake levels for particular species or categories [104]. These intake levels are determined by the maximum content of vitamin A authorized for use in feed. However, since feed additive legislation is periodically adjusted, it is advisable to constantly check for local updates and possible changes in the allowed applications. Notably, the occurrence of hypervitaminosis A in animals is highly improbable and has not been reported in practical farming or husbandry scenarios, thanks to the stringent regulation of vitamin A levels in animal feed [140]. The EFSA FEEDAP Panel has determined that the current practical levels of vitamin E used in animal production are safe for all animal species. The data on hypervitaminosis E are not consistent enough to establish a maximum safe content for vitamin E in feedstuffs based on animal safety [141].

## 7. Challenges in Applying Liquid Formulations across Animal Species

Administering liquid vitamins A and E to livestock and pets presents several challenges across different species. These challenges primarily involve ensuring consistent intake and maintaining vitamin stability.

### 7.1. Cattle

One major challenge with cattle is ensuring the uniform distribution of liquid vitamins. In large herds, individual animals may consume varying amounts of water or feed, leading to inconsistent vitamin intake [142]. This variability can affect the overall effectiveness of supplementation, particularly when vitamins are added to drinking water or mixed feeds. Even if vitamin E acetate is stable, supplementing liquid vitamin A via feed poses challenges due to its instability and susceptibility to oxidation, leading to significant degradation and loss of efficacy [143]. Furthermore, interactions with other feed components and inconsistent distribution can undermine the effectiveness of the supplementation. Supplementing vitamin A via water is preferred, as it allows for the use of specially stabilized formulations designed to maintain the vitamin’s potency [2].

A more precise and dependable method for vitamin supplementation is through injectable administration [144]. This approach allows for the accurate dosing of vitamins to individual animals, ensuring that each receives the exact amount required. However, injectable supplementation tends to be more labor-intensive and can cause greater stress to the animals compared to other methods [145]. It often necessitates the presence of a veterinarian or an animal health professional, which can increase both the complexity and cost of the supplementation process.

### 7.2. Swine

The accurate dosing of liquid vitamins through dry or liquid feed can be particularly challenging in automated feeding systems for swine, where precise measurement is crucial to prevent over- or under-supplementation. These systems must ensure that the correct amount of vitamins is evenly distributed, which can be difficult due to variations in feed consumption among individual animals [146]. An alternative method is to administer vitamins through drinking water. However, similar to the challenges observed in cattle, this approach can lead to inconsistent vitamin intake because individual animals may consume different amounts of water.

In contrast, injection supplementation offers a more controlled method for administering vitamins. This technique allows for precise dosing, ensuring that each animal receives the exact amount of vitamins needed [144]. By targeting individual animals, injections eliminate variability in intake and guarantee that each animal gets the specified dosage, thereby enhancing the effectiveness of the supplementation.

### 7.3. Poultry

In domestic fowl, liquid vitamin supplements are typically administered through drinking water to facilitate consistent intake across the flock. A significant challenge with this method is ensuring that all birds receive an adequate amount of the vitamins, particularly in large flocks. Variability in individual water consumption can lead to uneven distribution of the micronutrients. Additionally, the stability of liquid vitamins in water can be influenced by factors such as water temperature and pH, necessitating adjustments in both the formulation of the supplements and the administration practices [147].

Injection methods are generally impractical for large flocks due to their labor-intensive nature and time constraints [2]. However, they are occasionally employed for larger fowl, such as ostriches, or for high-value birds within breeding flocks, where precise dosing and targeted supplementation are critical.

### 7.4. Pets

For pets, the primary challenge in administering liquid vitamins is ensuring their acceptance and consumption. Pets can be sensitive to changes in taste or smell, which may lead to reluctance in consuming the supplement when added to their feed or water [148]. Additionally, achieving the correct dosage can be difficult if the pet is particular about flavors or if the liquid formulation is unappealing.

To address these challenges, one effective approach is to consult with a veterinarian. A veterinarian can provide precise dosing through oral administration techniques or, if necessary, by injection [149]. This method ensures that the pet receives the correct amount of the supplement and may improve adherence to the prescribed regimen. Consistent administration, whether at home or through veterinary guidance, is crucial for realizing the intended health benefits of the vitamin supplements.

Thus, the effective administration of liquid vitamins A and E to livestock and pets presents notable challenges, including ensuring a consistent intake, maintaining vitamin stability, and managing the logistics of supplementation. While injectable methods offer precise dosing and mitigate intake variability, they often involve a higher labor and cost, underscoring the need for careful consideration of each method’s practicality and effectiveness.

## 8. Perspectives and Future Directions

Liquid forms of vitamins A and E have attracted significant interest in veterinary practice due to their potential benefits and ease of use. These essential micronutrients play crucial roles in animal health, including functions such as vision, immune response, reproductive health, and overall well-being. In the veterinary community, the use of liquid vitamin supplements is common, serving both preventive and therapeutic purposes [11].

Deficiencies in vitamins A and E can lead to various health problems in animals, from stunted growth and reproductive issues to compromised immune function [2,33,84,89]. Liquid formulations provide a convenient and effective way to address these deficiencies, especially when oral supplementation through water or parenteral administration is preferred. Their improved bioavailability ensures optimal micronutrient utilization, speeding up recovery from deficiency disorders.

Despite the previously mentioned challenges in using liquid formulations for animals, one of the key advantages of liquid vitamin supplements remains their ease of administration compared to dry forms. Unlike solid forms, liquids are easier to give, particularly to animals that resist oral medication or have difficulty swallowing [134,135]. Liquid supplements allow for precise dosing and can be easily mixed into water or directly administered into the mouth, making them more palatable and increasing compliance among animals [150].

Furthermore, liquid vitamin A and E supplements offer versatility in their use, allowing for tailored treatment regimens to meet the specific needs of individual animals. They can be used preventively to prevent deficiencies or therapeutically to address existing health issues, giving veterinarians flexibility in their treatment approach [103].

Looking ahead, the future of liquid vitamin A and E supplementation in veterinary practice holds promise for further improvement and innovation. Future research may focus on optimizing formulations to enhance stability, palatability, and bioavailability. This could involve developing new delivery systems or adding additives to improve the taste and texture, making supplements more appealing to animals [151].

Additionally, advances in veterinary medicine may lead to targeted therapies using liquid vitamin A and E supplements [136,152,153]. Tailored to specific animal species, breeds, or health conditions, these therapies could provide precise and effective treatment strategies, improving outcomes for animal patients.

Moreover, the growing field of nutrigenomics offers the potential for precision feeding in veterinary medicine for pets [67]. Future research may explore the interaction between genetic factors and vitamin requirements in animals, paving the way for targeted supplementation protocols based on individual genetic profiles.

As our understanding of the effects of liquid vitamin A and E supplements continues to evolve, there will be an increasing emphasis on evidence-based practice in veterinary medicine. Rigorous animal trials will be crucial to establish the efficacy and safety of these supplements, enabling veterinarians and animal health professionals to confidently incorporate them into standard treatment protocols.

## 9. Conclusions and Recommendations

This review highlights the essential role of liquid vitamin A and E supplements in veterinary and livestock production, emphasizing their advantages over traditional forms. The synergistic effects of these vitamins in maintaining cellular health and overall physiological well-being have been discussed, along with their benefits for ocular and immune system health, growth, and reproductive health.

Liquid supplements offer enhanced bioavailability and ease of administration. The bioavailability of vitamin A varies with administration methods, with direct bloodstream injection achieving the highest levels.

To advance the field of animal health and enhance livestock productivity and welfare, several key recommendations should be pursued. First, optimizing formulations of liquid vitamins A and E is crucial to improving their stability, palatability, and bioavailability for different animal species. This can ensure that the supplements are both effective and well received by the animals. Next, exploring targeted therapies by investigating individualized vitamin delivery approaches, based on genetic and individual animal profiles, could lead to more tailored and effective supplementation strategies. Additionally, conducting rigorous trials to establish the efficacy and safety of these liquid supplements is essential, adhering to evidence-based practices to ensure reliable results. Standardizing protocols for integrating liquid supplements into veterinary practices is also important, including considerations for dosages, administration routes, and potential drug interactions. By implementing these recommendations, we can enhance animal health, reproductive outcomes, growth, and overall well-being. Continued research and adherence to evidence-based practices will contribute to more effective management strategies and improved animal care.

## Figures and Tables

**Table 1:** Estimated adequate vitamin A and E intake in dairy cattle [17,58].

Committee	Adequate Intake of Vitamin A, IU/kg BW			
	Growing Heifers	Dry Cows	Lactating Cows, (<35 kg milk/d)	Lactating Cows, (>35 kg milk/d)
NASEM [58]	110	110	110	n/a
NASEM [17]	110	110	110	110 + (1000 × (milk-35))
	**Adequate Intake of Vitamin E, IU/kg BW**			
NASEM [58]	0.8	1.6	0.8	n/a
NASEM [17]	0.8	1.6	0.8 (3) *	0.8 (3) *

* Increased to 3 IU/kg BW for pre-fresh cows (2–3 weeks before parturition).

**Table 2:** Estimated adequate vitamin A and E intake in beef cattle [57,59].

Committee	Adequate Intake of Vitamin A, IU/kg DM Intake		
	Growing and Finishing	Gestating Cows	Lactating Cows
NASEM [57]	2200	2800	3900
NASEM [59]	2200	2800	3900
	**Adequate Intake of Vitamin E, IU/kg DM Intake**		
NASEM [57]	15–60	-	-
NASEM [59]	15–60	15–20	40–60

**Table 3:** Vitamin A and E dietary recommendations for monogastric species.

Animal Species	Vitamin A Recommendation, IU/kg Feed	Vitamin E Recommendation, IU/kg Feed	Source
Broilers	10,000–13,000	55–80	[71,72]
Laying hens	8000–13,000	20–30	[73,74]
Turkeys	5000–12,500	20–100	[75,76]
Broiler breeders	10,000–13,000	100	[77,78]
Piglets	8000–16,000	84–150	[79,80,81,82]
Finishing gilts and barrows	4000–10,000	33–80	[79,80,81,82]
Lactating gilts and sows	9920–14,000	60–100	[79,80,81,82]
Dogs	8000–12,000	80–120	[83]
Cats	15,000–25,000	100–150	[83]

**Table 4:** Diseases associated with vitamin E deficiency in different animal species [3].

Disorder	Animal Model	Compromised Organ/Tissue
Immune deficiency	Chick, pig	Mononuclear phagocyte system
Myopathic disorders	Rabbit, duck, lamb, calf, turkey, chicken	Heart, skeletal muscles, gizzard
Reproductive dysfunction		
embryonic apoptosis	Hen, turkey, cow	Embryonic circulatory system
infertility (male)	Rooster, rabbit	Testes
Kidney, pancreas, liver, brain, blood		
necrobiosis	Pig	Liver
erythrocyte hemolysis	Chick, calf	Red blood cells
hypoproteinemia	Chick, turkey	Ricin
cerebral softening	Chick, duckling	Encephalon
hemorrhagic diathesis	Chick, turkey	Vascular system
nephrosis	Mink, rat	Renal tubular
yellow fat disease	Pig	Adipose tissue

**Table 5:** Impacts of neonatal vitamin A administration on cattle growth performance * (adapted from Harris et al. [24]).

Parameter	0 IU (n = 9)	150,000 IU (n = 7)	300,000 IU (n = 9)	SE
Birth to weaning				
Birth weight, kg	35.1	35.6	35.6	0.58
Average daily gain, kg/d	0.88 ^b^	0.98 ^a^	1.00 ^a^	0.02
Backgrounding				
Weaning weight at d 210, kg	219.2	248.7	246.0	5.98
Gain/feed ratio, kg	0.149	0.153	0.156	0.031
Finishing				
Weight at 308 d, kg	312.1 ^b^	333.0 ^ab^	339.7 ^a^	8.65
Feed/gain ratio, kg	4.35	4.76	4.79	0.13

* Angus steer calves (n = 30) were randomly allocated to three treatment groups at birth, receiving 0, 150,000, or 300,000 IU of vitamin A at both birth and one month of age. ^a,b^ Mean values within a row with no common superscript differ significantly (*p* < 0.05).

## Data Availability

Not applicable.

## Abbreviations

AKT	Serine-threonine kinase B
ATRA	All-trans-retinoic acid
BHA	Butylhydroxyanisole
BHT	Butylhydroxytoluene
BW	Body weight
DM	Dry matter
EFSA	European Food Safety Authority
EMA	European Medicines Agency
FEEDAP	The Panel on Additives and Products or Substances used in Animal Feed
FRAP	Ferric Reducing Ability of Plasma
i.m.	Intramuscular
IBV	Infectious bronchitis virus
ID3	DNA-binding inhibitor 3
IgG	Immunoglobulin G
IU	International Unit
KEAP1	Kelch-like ECH-associated protein 1
mTOR	Mammalian Target for Rapamycin
MYF5	Myogenic Factor 5 (a protein coding gene)
MYOD	Myoblast Determination Protein
MYOG	Myogenin
NASEM	The National Academies of Sciences, Engineering, and Medicine
NFκB	Nuclear Factor kappa-light-chain-enhancer of activated B cells
NRF2	Nuclear Factor Erythroid 2–related factor 2
PAX7	Paired Box 7 (a protein-coding gene)
PEG	Polyethylene glycol
RFM	Retained fetal membranes
s.c.	Subcutaneous
UV	Ultraviolet
40BHLHE40	Class E basic helix-loop-helix protein
